# Agreement between the Apple Series 1, LifeTrak Core C200, and Fitbit Charge HR with Indirect Calorimetry for Assessing Treadmill Energy Expenditure

**DOI:** 10.3390/ijerph16203812

**Published:** 2019-10-10

**Authors:** Peng Zhang, Ryan Donald Burns, You Fu, Steven Godin, Wonwoo Byun

**Affiliations:** 1 Department of Physical Education, East Stroudsburg University, East Stroudsburg, PA 18301 USA; pzhang@po-box.esu.edu; 2 Department of Health, Kinesiology, and Recreation, University of Utah, Salt Lake City, UT 84112, USA; won.byun@utah.edu; 3 School of Community Health Sciences, University of Nevada Reno, Reno, NV 89557, USA; youf@unr.edu; 4 Department of Family and Preventive Medicine, University of Utah, Salt Lake City, UT 84112, USA; Steven.godin@utah.edu

**Keywords:** activity trackers, physical activity, wearable technology

## Abstract

The purpose of this study was to examine agreement in energy expenditure between the Apple Series 1 Watch, LifeTrak Core C200, and Fitbit Charge HR with indirect calorimetry during various treadmill speeds in young adults. Participants were a sample of college-aged students (mean age = 20.1 (1.7) years; 13 females, 17 males). Participants completed six structured 10-minute exercise sessions on a treadmill with speeds ranging from 53.6 m·min^−1^ to 187.7 m·min^−1^. Indirect calorimetry was used as the criterion. Participants wore the Apple Watch, LifeTrak, and Fitbit activity monitors on their wrists. Group-level agreement was examined using equivalence testing, relative agreement was examined using Spearman’s rho, and individual-level agreement was examined using Mean Absolute Percent Error (MAPE) and Bland-Altman Plots. Activity monitor agreement with indirect calorimetry was supported using the Apple Watch at 160.9 m·min^−1^ (Mean difference = −2.7 kcals, 90% C.I.: −8.3 kcals, 2.8 kcals; MAPE = 11.9%; r_s_ = 0.64) and 187.7 m·min^−1^ (Mean difference = 3.7 kcals, 90% C.I.: −2.2 kcals, 9.7 kcals; MAPE = 10.7%; r_s_ = 0.72) and the Fitbit at 187.7 m·min^−1^ (Mean difference = −0.2 kcals, 90% C.I.: −8.8 kcals, 8.5 kcals; MAPE = 20.1%; r_s_ = 0.44). No evidence for statistical equivalence was seen for the LifeTrak at any speed. Bland-Altman Plot Limits of Agreement were narrower for the Apple Series 1 Watch compared to other monitors, especially at slower treadmill speeds. The results support the utility of the Apple Series 1 Watch and Fitbit Charge HR for assessing energy expenditure during specific treadmill running speeds in young adults.

## 1. Introduction

Statistics from the World Health Organization showed that physical inactivity is one of the major leading causes of human mortality, with approximately 25% of adults failing to meet recommended guidelines for physical activity (PA) [1]. Being able to monitor individual’s PA plays an important role on PA promotion and weight-management initiatives [2]. In recent years, both consumers and researchers have increased usage of wrist-worn wearable PA technology, which provide an opportunity for people to self-monitor their PA behaviors [3,4,5]. Wearable technologies, such as Fitbit and Apple Watch, provide a direct estimate of steps, PA time, and energy expenditure (EE) under various conditions [6,7,8]. A World-Wide Survey of Fitness Trends has ranked wearable technology a top fitness trend from 2015 to 2016 as more than 42 million wearable devices were sold in 2013 [9,10]. Public health professionals indicated that these devices may serve as a more cost-effective and appealing intervention method for behavior-change applications [11,12].

With the growing usage of wearable activity monitors, a considerable amount of research has been conducted to examine the validity of commercially available activity monitors for estimating PA levels (e.g., steps and EE) across different age groups [13,14,15]. Among these monitors, the most frequently studied are the Fitbit trackers, Sensewear Armband (Bodymedia, Pittsburgh, PA, USA), and Samsung Galaxy Gear (Samsung, Seoul, South Korea). Most studies that tested the validity of activity monitors have used indirect calorimetry or doubly labeled water as the criterion measure, while some used ActiGraph (ActiGraph, Pensacola, FL, USA)—a validated physical activity monitor—for the comparison [16,17]. The studies yielded mixed results, indicating inconsistent EE estimation from different types of monitors for various physical activities under controlled, semi-structured, and free-living conditions. Indeed, Sasaki’s research (2015) revealed that Fitbit HR significantly underestimated EE calculated from Oxycon Mobile indirect calorimetry and yielded large variability across nine activities, while a study from Lee et al. [18] reported that BodyMedia Fit and Fitbit Zip provided acceptable validity of EE among a group of young adults across four different types of physical activities. Work from Bai et al. [6] showed that the Apple Watch 1 yielded higher accuracy than the Fitbit Charge HR for assessing overall EE during aerobic exercise among 18 to 65 year-old adults. Chowdhury and colleagues [14] compared Apple Watch to Fitbit HR on EE estimate of a 24-h physical activity in free-living conditions by using BodyMedia Core as the criterion measure. Their results concluded that commercial monitors were not equivalent to the research-grade counterparts, even though Apple Watch performed a more consistent estimate on 24-h EE. Using different algorithms to estimate EE in the wearable devices may serve as a major reason why some devices are more accurate than others. For example, companies have applied distinctive machine learning techniques to enable their classifications of underlying physical activity patterns. The equations developed for calculating EE varied among different devices [19]. Consequently, additional research is warranted to compare results from various types of wearable fitness devices with different populations, activities, and criterion measures for validity verification purposes.

The current literature suggests that despite the widespread consumption and potential applications of wearable devices, there is still limited understanding regarding the validity of standard accelerometry-based activity monitors for assessing EE [3,6,13]. It is recommended that additional research is warranted to compare results with other commercial and research monitors, with different activities, and sample populations [3,6]. Therefore, the purpose of this study was to examine the agreement in estimated energy expenditure among the Apple Series 1 Watch, LifeTrak Core C200, and Fitbit Charge HR with indirect calorimetry during various treadmill speeds in young adults. The present study adds new information to the literature by formally evaluating the validity of three different consumer-based, activity monitoring technologies under a controlled (lab) condition, with estimates of EE from an indirect calorimetry test as the criterion measure.

## 2. Materials and Methods

### 2.1. Participants

Participants were a convenience sample of thirty young adults (mean age = 20.1 (1.7) years; 13 females, 17 males). The inclusion of the participants was based on the following screening criteria: (1) a regular participation in exercise in the past three months (>3 times per week with 30-min minimum on each day’s activity) and (2) participants responding ‘No’ to all Physical Activity Readiness Questionnaire (PAR-Q) questions. We also excluded individuals who were prescribed medication from the study. All participants signed a consent form during recruitment and the University Institutional Review Board approved the research protocol prior to the start of the study. The study is complied with the Declaration of Helsinki.

### 2.2. Instrumentation

Apple Watch Series 1 (Apple Inc., Cupertino, CA, USA) is a wrist-worn smart watch compatible with an iPhone 5^®^ or more updated models. This study selected three 38 mm Apple Watch Series 1 (AWS1), which were labeled as AWS1a, AWS1b, and AWS1c for data collection purposes. Each AWS1 device included a workout application that tracked walking or running activities with total calorie estimates. The total calorie estimates were summed across active and resting calories. All the AWS1 watches were placed on the right wrist of the participants in accordance with the manufacturer’s recommendations and remained in this anatomical location throughout testing. Each AWS1 device was synced with an iPhone and updated with participant’s information such as height (ft), weight (lb), and sex prior to the walking and running bouts.

Fitbit HR (Fitbit Inc., San Francisco, CA, USA) is a wrist-worn activity tracker compatible with both Android and iOS platforms that have Bluetooth technology for data syncing with a Fitbit application. Activity tracking features contain calories, steps, and distance. Fitbit was worn on the left wrist of each participant and was synced to an iPhone to obtain their physical characteristics.

LifeTrak (LifeTrakUSA Inc., Newark, NJ, USA) is a wrist-worn activity tracker capturing a set of exercise-related measures, such as steps, calories, distance, and heart rate. LifeTrak was consistently placed on the left wrist of participants and the device did not require synchronization with smart phones for individual bio information input. Because of high reliability of the estimates between dominant and non-dominant arm wearing and the fact that some of our participants were unable to wear all the devices on one arm, we requested all participants to wear the devices in the same body location and same order throughout the testing [20].

A metabolic cart (TrueOne 2400, Parvo Medics Inc., Sandy, UT, USA) was utilized to measure oxygen consumption and carbon dioxide production during the physical activity protocol and served as the criterion measure for EE. The sampling rate was 60 s and data were collected every minute. Prior to each testing session, the oxygen and carbon dioxide analyzers were calibrated according to the manufacturer’s instructions. The calibration included room air calibration, reference gas calibration using 16% and 5% carbon dioxide, and flow calibration using a 3.00 L syringe.

### 2.3. Procedures

Participants completed four testing sessions recommended as a standard exercise assessment protocol published by Abel and colleagues: (1) body composition and demographic data, (2) Resting Metabolic Rate (RMR) and familiarization, (3) a walking session, and (4) a running session (Abel et al., 2011). All the testing was performed in a University’s Applied Physiology Laboratory, while the walking and running sessions were randomly assigned to the participants.

Whole-body plethysmography was used as a method to assess participants’ body composition (BOD POD Body Composition System, COSMED USA, Inc., Concord, CA, USA) at the beginning of a testing day, a standardized calibration was performed to enhance accuracy. In addition, participants’ body height, body weight, body composition, and exercise history were collected in this session. Weight was measured to the nearest 0.1 kg and height was measured to the 0.1 cm using a stadiometer (Detecto 439, Web City, MT, USA). Both height and weight were measured using standardized procedures with participants in light clothes and without shoes.

Participants reported to the university laboratory between the hours of 6 a.m. and 9 a.m., following a 12 h fast and 24 h refrainment of stimulants (i.e., caffeine). The resting metabolic rate measure began as participants were asked to relax in a reclined position in a dimly lit room for 20 min. After the initial 20-min rest, participants’ expiratory gases were collected for 22 min. Expiratory gas collection continued for an additional 22 min, while the expiratory gas data in the first two minutes were dismissed. The remaining 20 min of expiratory gas data were used to calculate resting metabolic rate.

The participants performed a modified walking and running trial on the treadmill as a familiarization at speeds of 80 m∙min^−1^ and 161 m∙min^−1^, respectively. Each trial lasted for five minutes with a two-minute resting interval for simulating the real testing environment. All participants were required to wear all the monitors on their wrists during the familiarization trials. Participants received training on safety procedures regarding stepping on and off the moving treadmill.

Participants completed two sets of three 10-min treadmill walking and running trials. The three walking trials were at speeds of 53.6, 80.5, and 107.3 m·min^−1^, while the running trials were at speeds of 134.1, 160.9, 187.7 m·min^−1^ (Abel et al., 2011). A three-minute washout period was used between each trial, which we expected to allow recovery to an approximately resting metaboic rate. For ensuring steady-state energy expenditure, only the last seven minutes of indirect calorimetry data from each 10-min treadmill trials were selected for analysis for all participants; this allowed each participant three minutes to reach steady-state VO_2_.

### 2.4. Statistical Analysis

Differences between sexes on all descriptive characteristics were examined using independent *t*-tests. Effect sizes were calculated using Cohen’s d, with d < 0.20 indicating a small effect size, d = 0.50 indicating a medium effect size, and d > 0.80 indicating a large effect size [21]. Equivalence testing was employed to test the null hypothesis that there is no equivalence (non-equivalence) between the criterion (indirect calorimetry) and surrogate (activity monitors) group energy expenditure means. It has recently been advised that equivalence testing be used over more traditional analytical approaches using the general linear model (e.g., *t*-tests, ANOVA) to determine group-level agreement between or among assessments of PA from varying methods [22]. A ±10% of the mean estimates from the criterion measure was employed as the specified equivalence zone and it was compared with the 90% confidence intervals of the estimates from activity monitors tests. If the calculated 90% Confidence Intervals of the estimates from activity monitors fell entirely within the equivalence zone of the criterion measure, the null hypothesis was rejected and it was deemed that estimates were statistically equivalent at the *p* < 0.05 level.

To quantify relative agreement, Spearman’s rho correlation coefficients (r_s_) were calculated between estimated and measured energy expenditure for each device against indirect calorimetry across all treadmill speeds. Spearman’s rho was used instead of the Pearson product–moment correlation due to the small sample size. Spearman’s rho correlation coefficients were interpreted as high if r_s_ > 0.70, moderate if r_s_ = 0.50 to 0.70, low if r_s_ = 0.30 to 0.50, and negligible if r_s_ = 0.00 to 0.30 [23]. Individual-level agreement was assessed using the mean absolute percent error (MAPE) and Bland–Altman Plots. MAPE was calculated and Bland–Altman Plots were utilized, using indirect calorimetry as the criterion, for all activity monitors across all treadmill speeds. Results were graphically derived to visually depict the variability in MAPE across treadmill speeds and activity monitors. Differences in absolute percent error were correlated with BMI and mean differences in absolute percent error between sexes were examined using unpaired *t*-tests to determine if these characteristics explained any of the observed bias between measured and estimated energy expenditure. Bland–Altman plots involved plotting the average of estimated and measured energy expenditure on the x-axis and the difference in estimated and measured energy expenditure on the y-axis. From the Bland–Altman plots, mean bias (mean differences) were calculated along with the 95% Limits of Agreement. All analyses had an alpha level of *p* < 0.05 for statistical significance and were carried out using the STATA v14.0 statistical software package (StataCorp LLC, College Station, TX, USA).

## 3. Results

The descriptive statistics are reported in Table 1. Males had lower percent body fat (mean difference = −10.9%, *p* = 0.009, d = 1.73) and higher percent fat-free mass (mean difference = 10.9%, *p* = 0.008, d = 1.73) and BMI (mean difference = 2.1 kg/m^2^, *p* = 0.008, d = 0.81) compared to females. There was no significant difference between sexes in age.

Table 2 presents the results from the equivalence testing in addition to Spearman’s rho scores. Equivalence was statistically supported, using indirect calorimetry energy expenditure as the criterion, for the Apple Watch at 160.9 m/min and 187.7 m/min and for the Fitbit at 187.7 m/min (*p* < 0.05). No evidence for statistical equivalence was seen for the LifeTrak at any speed. In general, the trends were for significant error at the slower walking speeds and improved error and better agreement when the participants were engaged in the faster treadmill running speeds. This phenomenon is reflected in the mean difference and Spearman’s rho scores across the various treadmill speeds and activity monitors. Spearman’s correlations ranged from moderate-to-strong across most treadmill speeds (*p* < 0.05), except for the LifeTrak, where all correlations were not statistically significant (see Table 2).

**Figure 1:**
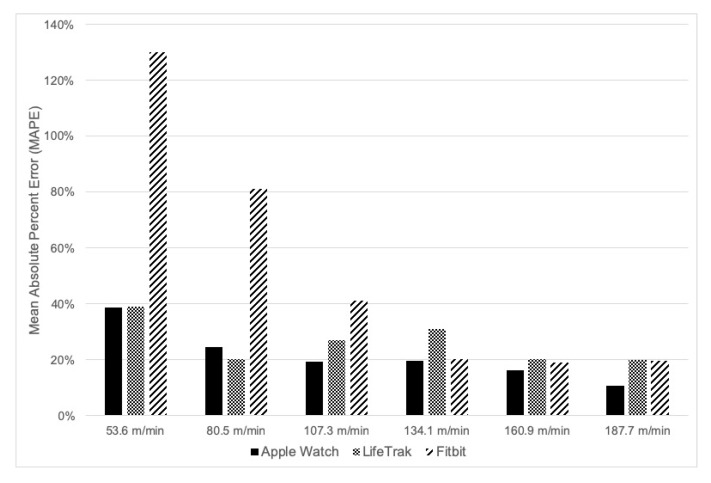
Mean Absolute Percent Error scores across treadmill speeds.

Figure 1 displays the MAPE scores across treadmill speeds for each activity monitor. MAPE ranged from as high as 130% for the Fitbit at a walking speed of 53.6 m·min^−1^ to as low as 10.7% for the Apple Watch at a running speed of 187.7 m·min^−1^. Like the equivalence scores, the trend was high MAPE at slower treadmill speeds and lower MAPE at faster treadmill speeds compared to indirect calorimetry. At treadmill speeds of 160.9 m·min^−1^ to 187.7 m·min^−1^, MAPE ranged from approximately 10% to 20% across all activity monitors, with the Apple Watch slightly outperforming the LifeTrak and Fitbit (see Figure 1). Absolute percent error scores correlated with BMI using the LifeTrack at 134.1 m/min (r = −0.47, *p* = 0.008), suggesting a negative association between BMI and MAPE (higher MAPE, lower BMI). However, no other significant associations were found between BMI and MAPE for any of the other devices at any of the other speeds. Concerning sex differences in absolute percent error, females had higher error than males using the Apple Watch(a) at 187.7 m/min (mean differences = 10.7%, *p* = 0.03, d = 0.84), males had greater absolute percent error than females using the LifeTrak at 134.1 m/min (mean differences = 30.0%, *p* < 0.001, d = 1.11) and at 160.9 m/min (mean differences = 16.0%, *p* = 0.028, d = 0.81). No other sex differences were found for any other devices at any other speed.

Table 2 also reports the results from the Bland–Altman plots. The 95% Limits of Agreement were relatively wider for the Fitbit compared to all other devices. Apple Watches tended to have narrower Limits of Agreement compared to the LifeTrak and Fitbit, especially at slower speeds. The percentage of scores outside the Limits of Agreement ranged from 3.33 to 10.0%.

## 4. Discussion

The purpose of this study was to examine the agreement in estimated EE among the Apple Series 1 Watch, LifeTrak Core C200, and Fitbit Charge HR with indirect calorimetry during various treadmill walking and running speeds in a sample of young adults. The results indicated that the Apple Watch and LifeTrak outperformed Fitbit in estimating EE, relative to indirect calorimetry, at slow walking speeds, and the Apple Watch marginally outperformed the LifeTrak and Fitbit at faster treadmill running speeds. Instrument EE statistical equivalence with indirect calorimetry was found using the Apple Watch at 160.9 m·min^−1^ and 187.7 m·min^−1^ and the Fitbit at 187.7 m·min^−1^. The LifeTrak did not display statistical equivalence with indirect calorimetry at any treadmill speed. Taken together, these results suggest that the Apple Watch and Fitbit may be valid instruments for estimating EE at high-intensity PA; however, the utility of these devices is questionable at lower-intensity PA. Also, based on the current findings, the LifeTrak activity monitor cannot be recommended as a valid tool to estimate EE.

Noah and colleagues [24] compared among Fitbit, Actical accelerometer (Phillips Respironics), and indirect calorimetry and concluded that the Fitbit activity tracker was generally reliable and valid as compared to standard research-grade accelerometers [24]. Specifically, Fitbit could accurately estimate EE under the settings of walking (94 m·min^−1^), and stepping when no incline was incorporated, but was slightly less accurate for jogging (147 m·min^−1^) and incline walking (94 m·min^−1^). In another study, Sasaki et al. found that Fitbit systematically underestimated EE during various activities under both free-living (e.g., office locomotion activities; sport activities; house activities) and lab (walking at 80 m·min^−1^ and 107 m·min^−1^, and ground-level jogging at 147 m·min^−1^) settings, while Fitbit demonstrated a relative reliable estimation when the participants were walking on a flat surface [8]. In a recent study, Bai and colleagues [6] investigated the validity of EE between Apple Watch series 1 and Fitbit Charger HR among 39 adults under different activities (20 min of sedentary activity, 25 min of aerobic exercise, and 25 min of light-intensity physical activity), the indirect calorimetry was used as the criterion. The results of the study showed that both Apple Watch 1 devices demonstrated better accuracy than Fitbit Charge HR on estimating energy expenditure, which was similar with the findings of the current study.

Previous literature has demonstrated the acceptable validity of the earlier Fitbit trackers for estimating EE when compared with indirect calorimetry, included Fitbit One, Fitbit Zip, and Fitbit Flex [25,26]. The present study is in line with three other recent studies that reported Fitbit products had relatively low validity in estimating EE [3,26,27]. According to Bai et al. [6], a plausible explanation might be that the EE prediction algorithms process in Fitbit Charger HR has not been fully debugged, it is still unclear whether energy expenditure prediction algorithms calibrations were applied to directly incorporate heart rate data in the estimations. Another surprising finding of the study showed that the devices tended to be less valid when the exercise intensity became lower. More measurement errors seem to take place as exercise intensity diminishes. It is uncertain how and why the devices tended to overestimate or underestimate EE in a low-intensity exercise setting. This result may be due to various designs of the hardware and software in the devices that have an impact on the sensitivity of the devices assessing user’s EE. Little is known in the literature about this situation and more research is recommended on this area.

Unlike the Apple Watch and Fitbit, the LifeTrak activity monitor is not used as often in validation studies or in studies requiring objective physical activity surveillance. LifeTrak Core C200 is a wearable device that can be worn on the upper or lower arm, wrist or ankle and measures heart rate and heart rate variability during activity, rest, and sleep. Based on the measured heart rate, EE is estimated. A salient finding from the current study was that non-equivalence was found across all treadmill speeds using the LifeTrak. Although absolute mean differences were relatively small across the range of treadmill speeds, there was large inter-individual variability in the EE estimates and presence of bias across the range of EE when the participants were running on the treadmill at a speed of 187.7 m·min^−1^. Because of these results, it is difficult to recommend the LifeTrak Core C200 as a valid proxy for EE. To the authors’ knowledge this is the first study to examine the agreement and equivalency of the LifeTrak Core C200 with indirect calorimetry using a range of treadmill speeds. Because the LifeTrak can be worn on different peripheral locations on the body, future research should further explore the reliability and validity of the device at the different locations to determine the LifeTrak’s utility in research and in clinical practice. Because the LifeTrak can be worn on different peripheral locations on the body, future research should further explore the reliability and validity of the device at the different locations to determine the LifeTrak’s utility in research and in clinical practice.

There are limitations to this study that must be discussed before the results can be generalized. First, the sample consisted of college-aged students recruited from one University located in the Northeast US; therefore, it is questionable if the results can generalize to younger or older age groups. Second, agreement and equivalence were analyzed during walking and running on a treadmill; therefore, the psychometric properties of the Apple Watch, LifeTrak, and Fitbit during free-living leisure physical activity or during activities that are non-ambulatory in nature (e.g., swimming, resistance training, etc.) are unknown. This must be explored with additional research. Third, testing was across two days but the order was not counterbalanced; therefore, there could be testing effects that threaten the internal validity of the results. Fourth, it is worth noting that the results may not be generalized to newer devices due to rapid technology advancement. Finally, heart rate, steps, and resting metabolic rate all may influence energy expenditure predicted by the wearable monitors; however, these potential confounding variables were not controlled for in the analysis.

## 5. Conclusions

The Apple Watch and Fitbit are valid instruments for assessing energy expenditure during treadmill running in college-aged adults. Based on the agreement and equivalence scores, the LifeTrak cannot be recommended as a valid activity monitor for estimating energy expenditure at any treadmill speed. Despite these positive findings, the agreement and equivalence of all devices is questionable during slower walking speeds and more work needs to be conducted on these devices in free-living conditions and during activities that are non-ambulatory. Accuracy should also be tested in older age groups to establish the external validity of the Apple Watch, LifeTrak, and Fitbit to estimate energy expenditure. This is the first study to examine the psychometric properties of the Apple Watch, LifeTrak, and Fitbit for estimating energy expenditure at various treadmill walking and running speeds relative to an indirect calorimetry criterion. Given the popularity of the Apple Brand and Fitbit, this study supports the utility of these activity monitors for accurate activity monitoring in young adults during treadmill exercise of higher intensities.

## Figures and Tables

**Table 1:** Participant descriptive statistics (means and standard deviations).

	Total (*N* = 30)	Females (*n* = 13)	Males (*n* = 17)
Age (years)	20.1 (1.7)	20.8 (1.9)	19.8 (1.4)
Body Mass Index (kg/m^2^)	25.0 (2.7)	23.8 (2.6)	**25.8 ^†^ (2.5)**
Fat Mass (%)	16.7 (8.3)	**23.2 ^†^ (6.3)**	12.3 (6.3)
Fat-Free Mass (%)	83.3 (8.3)	76.8 (6.3)	**87.7 ^†^ (6.3)**

*Note:* bold and ^†^ denotes statistical differences between sexes, *p* < 0.05.

**Table 2:** Agreement scores in energy expenditure compared to indirect calorimetry across various treadmill speeds.

Activity Monitor	Treadmill Speed (m min^−1^)	Indirect Calorimetry EE (kcals)	Mean Bias (kcals)	90% C.I. of Estimates (kcals)	Equivalence Zone (kcals)	Spearman’s Rho	95% Limits of Agreement (kcals)
Apple Watch(a)	53.6	30.9	−10.8	−13.9 to −7.7	−3.1 to 3.1	**0.49 ^†^**	−30.5 to 8.9
	80.5	40.2	−9.0	−11.4 to −6.6	−4.0 to 4.0	**0.60 ^†^**	−24.3 to 6.3
	107.3	60.5	−4.1	−7.9 to −0.4	−6.1 to 6.1	**0.39 ^†^**	−27.7 to 19.5
	134.1	93.2	−11.4	−16.2 to 6.5	−9.3 to 9.3	**0.71 ^†^**	−42.3 to 19.4
	160.9	109.9	**−2.7 ^†^**	−8.3 to 2.8	−10.9 to 10.9	**0.64 ^†^**	−37.8 to 32.3
	187.7	126.9	**3.7 ^†^**	−2.2 to 9.7	−12.6 to 12.6	**0.72 ^†^**	−33.9 to 41.4
Apple Watch(b)	53.6	30.9	−15.2	−18.9 to −11.5	−3.1 to 3.1	**0.42 ^†^**	−38.6 to 8.1
	80.5	40.2	−13.0	−17.0 to −9.1	−4.0 to 4.0	**0.49 ^†^**	−38.0 to 12.0
	107.3	60.5	−9.3	−14.7 to −3.9	−6.1 to 6.1	**0.35 ^†^**	−43.3 to 24.6
	134.1	93.2	−17.7	−24.5 to −10.9	−9.3 to 9.3	**0.60 ^†^**	−60.6 to 25.1
	160.9	109.9	−11.9	−19.6 to −4.2	−10.9 to 10.9	**0.56 ^†^**	−60.4 to 36.7
	187.7	126.9	**−3.1 ^†^**	−10.1 to 3.9	−12.7 to 12.7	**0.68 ^†^**	−47.3 to 41.1
Apple Watch(c)	53.6	30.9	−2.3	−4.5 to −0.1	−3.0 to 3.0	**0.51 ^†^**	−16.0 to 11.5
	80.5	40.2	−3.3	−5.8 to −0.9	−4.0 to 4.0	**0.60 ^†^**	−18.8 to 12.2
	107.3	60.5	**2.0 ^†^**	−2.1 to 6.0	−6.1 to 6.1	**0.41 ^†^**	−23.7 to 27.6
	134.1	93.2	−16.9	−22.1 to −11.5	9.3 to 9.3	**0.64 ^†^**	−50.3 to 16.6
	160.9	109.9	−9.1	−15.3 to −3.0	−10.9 to 10.9	**0.64 ^†^**	−47.9 to 29.6
	187.7	126.9	**−5.5 ^†^**	−12.7 to 1.8	−12.7 to 12.7	**0.65 ^†^**	−51.1 to 40.1
LifeTrak	53.6	30.9	0.6	−5.5 to 6.6	−3.1 to 3.1	0.08	−37.9 to 39.0
	80.5	40.2	6.7	4.0 to 9.3	−4.0 to 4.0	0.15	−10.2 to 23.6
	107.3	60.5	10.0	4.4 to 15.7	−6.1 to 6.1	0.06	−25.2 to 45.3
	134.1	93.2	−16.9	−25.1 to −8.8	−9.3 to 9.3	0.33	−65.9 to 32.0
	160.9	109.9	−3.7	−12.0 to 4.6	−10.9 to 10.9	0.17	−56.5 to 49.1
	187.7	126.9	10.4	0.6 to 20.2	−12.6 to 12.6	0.17	−51.5 to 72.3
Fitbit	53.6	30.9	−37.0	−47.5 to −26.5	−3.1 to 3.1	0.21	−103.1 to 29.1
	80.5	40.2	−32.1	−36.1 to −28.2	−4.0 to 4.0	**0.63 ^†^**	−57.2 to −7.2
	107.3	60.5	−23.7	−30.1 to 16.2	−6.1 to 6.1	**0.36 ^†^**	−65.4 to19.1
	134.1	93.2	−12.2	−18.9 to −5.6	−9.3 to 9.3	**0.52 ^†^**	−54.3 to 29.8
	160.9	109.9	−9.2	−16.6 to −1.8	−11.0 to 11.0	**0.54 ^†^**	−55.8 to 37.4
	187.7	126.9	**−0.2 ^†^**	−8.8 to 8.5	−12.0 to 12.0	**0.44 ^†^**	−54.6 to 54.3

*Note:* Mean difference is (Criterion kcals—Activity Monitor kcals); EE stands for energy expenditure; Criterion is energy expenditure (kcals) measured from indirect calorimetry; 90% C.I. stands for the 90% Confidence Interval; Equivalence is denoted if the 90% C.I. falls completely within the equivalence interval; bold and ^†^ denotes statistical significance, *p* < 0.05; mean difference statistical significance denotes the null hypothesis rejection of non-equivalence.

## References

[1] World Health Organization 2018 Physical Activity Fact Sheet. http://www.who.int/mediacentre/factsheets/fs385/en/.

[2] Lee I.-M., Shiroma E.J. (2014). Using accelerometers to measure physical activity in large-scale epidemiological studies: Issues and challenges. Br. J. Sports Med..

[3] Bai Y., Welk G.J., Nam Y.H., Lee J.A., Lee J., Kim Y., Meier N.F., Dixon P.M. (2016). Comparison of consumer and research monitors under semi-structured settings. Med. Sci. Sports Exerc..

[4] Boudreaux B., Hebert E., Hollander D.B., Williams B.M., Cormier C.L., Naquin M.R., Gillan W.W., Gusew E.E., Kramer R.R. (2018). Validity of Wearable Activity Monitors during Cycling and Resistance Exercise. Med. Sci. Sports Exerc..

[5] Sperlich B., Holmberg H.C. (2017). Wearable, yes, but able…?: It is time for evidence-based marketing claims!. Br. J. Sports Med..

[6] Bai Y., Hibbing P., Mantis C., Welk G.J. (2018). Comparative evaluation of heart rate-based monitors: Apple Watch vs Fitbit Charge HR. J. Sports Sci..

[7] Grant P.M., Ryan C.G., Tigbe W.W., Granat M.H. (2006). The validation of a novel activity monitor in the measurement of posture and motion during everyday activities. Br. J. Sports Med..

[8] Sasaki J.E., Hickey A., Mavilia M., Tedesco J., John D., Keadle S.K., Freedson P.S. (2015). Validation of the Fitbit Wireless Activity Tracker for prediction of energy expenditure. J. Phys. Act. Health.

[9] Thompson W. (2015). Worldwide survey of fitness trends for 2016: 10th anniversary edition. ACSM Health Fit. J..

[10] Thompson W. (2016). Worldwide survey of fitness trends for 2017. ACSM Health Fit. J..

[11] Bassett D.R., Ainsworth B.E., Swartz A.M., Strath S.J., O’Brien W.L., King G.A. (2000). Validity of four motion sensors in measuring moderate intensity physical activity. Med. Sci. Sports Exerc..

[12] Welk G.J., Schaben J.A., Morrow J.R. (2004). Reliability of accelerometry-based activity monitors: A generalizability study. Med. Sci. Sports Exerc..

[13] Imboden M.T., Nelson M.B., Kaminsky L.A., Montoye A.H. (2018). Comparison of four Fitbit and Jawbone activity monitors with a research-grade ActiGraph accelerometer for estimating physical activity and energy expenditure. Br. J. Sports Med..

[14] Chowdhury E.A., Western M.J., Nightingale T.E., Peacock O.J., Thompson D. (2007). Assessment of laboratory and daily energy expenditure estimates from consumer multi-sensor physical activity monitors. PLoS ONE.

[15] Ryan J., Gormley J. (2013). An evaluation of energy expenditure estimation by three activity monitors. Eur. J. Sport Sci..

[16] Abel M.G., Hannon J.C., Sell K., Lillie T., Conlin G., Anderson D. (2008). Validation of the Kenz Lifecorder EX and ActiGraph GT1M accelerometers for walking and running in adults. Appl. Phys. Nutr. Metab..

[17] Manore M., Brown K., Houtkooper L., Jakicic J., Peters J., Edge M., Steiber A., Going S., Gable L.G., Krautheim A. (2014). Energy balance at a crossroads: Translating the science into action. Med. Sci. Sports Exerc..

[18] Lee J.-M., Kim Y., Bai Y., Gaesser G.A., Welk G.J. (2016). Validation of the SenseWear mini armband in children during semi-structure activity settings. J. Sci. Med. Sport.

[19] Lee J., Kim Y., Welk G. (2014). Validity of consumer-based physical activity monitors. Med. Sci. Sports Exerc..

[20] De Man M., Vanderploeg E., Aimers N., MacMahon C., Wise L., Parrington L. (2016). Validity and inter-device reliability of dominant and non-dominant wrist worn activity trackers in suburban walking. Sens. A J. Mind Brain Cult..

[21] Cohen J. (1988). Statistical Power Analysis for the Behavioral Sciences.

[22] Dixon P.M., Saint-Maurice P.F., Kim Y., Hibbing P., Bai Y., Welk G.J. (2018). A primer on the use of equivalence testing for evaluating measurement agreement. Med. Sci. Sports Exerc..

[23] Mukaka M.M. (2012). A guide to appropriate use of correlation coefficient in medical research. Malawi Med. J..

[24] Noah J.A., Spierer D.K., Gu J., Bronner S. (2013). Comparison of steps and energy expenditure assessment in adults of Fitbit Tracker and Ultra to the Actical and indirect calorimetry. J. Med. Eng. Technol..

[25] Lauritzen J., Muñoz A., Luis Sevillano J., Civit A. (2013). The usefulness of activity trackers in elderly with reduced mobility: A case study. Stud. Health Technol. Inform..

[26] Schneider M., Chau L. (2016). Validation of the Fitbit Zip for monitoring physical activity among free-living adolescents. BMC Res. Notes.

[27] Wallen M.P., Gomersall S.R., Keating S.E., Wisløff U., Coombes J.S. (2016). Accuracy of heart rate watches: Implications for weight management. PLoS ONE.

